# Retraction: RASSF8 downregulation promotes lymphangiogenesis and metastasis in esophageal squamous cell carcinoma

**DOI:** 10.18632/oncotarget.28882

**Published:** 2026-05-13

**Authors:** Lan Zhang, Jian-Hua Wang, Rong-Xin Liang, Shu-Ting Huang, Jing Xu, Lin-Jing Yuan, Long Huang, Yun Zhou, Xing-Juan Yu, Shao-Yun Wu, Rong-Zhen Luo, Jing-Ping Yun, Wei-Hua Jia, Min Zheng

**Affiliations:** ^1^Department of Gynecology, Guangzhou, P. R. China; ^2^Department of Pathology, Sun Yat-Sen University Cancer Center; Guangzhou, P. R. China; ^3^State Key Laboratory of Oncology in South China, Guangzhou, P. R. China; ^4^Collaborative Innovation Center for Cancer Medicine, Guangzhou, P. R. China; ^5^Cardiovascular Department, Second People’s Hospital of Guangdong Province, Guangzhou, P. R. China; ^6^Department of Oncology, The Second Affiliated Hospital, Nanchang University, Nanchang, P. R. China; ^*^These authors have contributed equally to this work

**This article has been retracted:** Oncotarget has concluded its investigation of this paper. Our findings identified several instances of internal duplication and overlaps with an unrelated paper from a different institution.

Specifically, the following issues were identified:

- Figure 2B: The wound healing assay panels for EC109/RASSF8-RNAi#1 and RASSF8-RNAi#2 at 48 h overlap.
- Figure 3A: The tube formation assay images for EC109/vector and RASSF8-RNAi#2, and EC109/RASSF8-RNAi#1 and KYES-410/RASSF8-RNAi#1, are duplicated. Furthermore, six out of eight panels in this figure were recycled in Figure 7 of a later publication [1].
- Figure 3B: The colony formation assay panels for EC109/vector and KYES-410/NC overlap.
- Figure 5D: In the western blot for KYES-410/p-ERK, the “vector” and “RASSF8” bands are duplicates of the two “RASSF8-RNAi#1” bands.

The authors did not respond to requests for comment or provide the original data. Consequently, the editorial decision has been made to retract this paper. Oncotarget has reached out to all authors to notify them of this retraction but has received no response.

Original article: Oncotarget. 2015; 6:34510–34524. 34510-34524. https://doi.org/10.18632/oncotarget.5923
